# A Two-Stage Interference Suppression Scheme Based on Antenna Array for GNSS Jamming and Spoofing

**DOI:** 10.3390/s19183870

**Published:** 2019-09-07

**Authors:** Jiaqi Zhang, Xiaowei Cui, Hailong Xu, Mingquan Lu

**Affiliations:** Department of Electronic Engineering, Tsinghua University, Beijing 100084, China; zhang-jq15@mails.tsinghua.edu.cn (J.Z.); xuhl07@163.com (H.X.); lumq@mail.tsinghua.edu.cn (M.L.)

**Keywords:** Global Navigation Satellite System (GNSS), anti-jamming, spoofing detection, spoofing mitigation, antenna array, subspace projection, Cyclic MUSIC algorithm

## Abstract

Jamming and spoofing are the two main types of intentional interference for global navigation satellite system (GNSS) receivers. Due to the entirely different signal characteristics they have, a few techniques can deal with them simultaneously. This paper proposes a two-stage interference suppression scheme based on antenna arrays, which can detect and mitigate jamming and spoofing before the despreading of GNSS receivers. First, a subspace projection was adopted to eliminate the high-power jamming signals. The output signal is still a multi-dimensional vector so that the spatial processing technique can be used in the next stage. Then, the cyclostationarity of GNSS signals were fully excavated to reduce or even remove the noise component in the spatial correlation matrix. Thus, the signal subspace, including information of the power and the directions-of-arrival (DOAs) of the GNSS signals, can be obtained. Next, a novel cyclic correlation eigenvalue test (CCET) algorithm was proposed to detect the presence of a spoofing attack, and the cyclic music signal classification (Cyclic MUSIC) algorithm was employed to estimate the DOAs of all the navigation signals. Finally, this study employed a subspace projection again to eliminate the spoofing signals and provide a higher gain for authentic satellite signals through beamforming. All the operations were performed on the raw digital baseband signal so that they did not introduce additional computational complexity to the GNSS receiver. The simulation results show that the proposed scheme not only suppresses jamming and spoofing effectively but also maximizes the power of the authentic signals. Nonetheless, the estimated DOA of spoofing signals may be helpful for the interference source positioning in some applications.

## 1. Introduction

With the extensive application of global navigation satellite systems (GNSS) in both military and civilian fields, the research of navigation countermeasure technology has gained more and more attention. Due to the inherent weakness of the satellite navigation systems, GNSS receivers are susceptible to both intentional and unintentional interference [1,2]. Jamming and spoofing are the two main kinds of intentional interference.

A jammer transmits high-power signals to the target receiver, which is very easy to implement because the power of the satellite signal reaching the ground is weak (about 20–30 dB below the thermal noise). It can degrade the carrier to noise ratio (C/N_0_) performance of the victim receiver or even put it into an “unlock” state [3]. Many relatively mature technologies can suppress this type of interference [4,5,6,7]. Among them, spatial processing based on an antenna array is considered as the most effective one. It can shape the reception beam pattern of the antenna array to form nulls toward jamming sources, thus the interferences are suppressed [8,9].

Spoofing is a more insidious and damaging interference that aims to mislead the target GNSS receiver to generate an erroneous position and timing solutions without awareness [10]. It can be realized by using a signal generator to counterfeit GNSS signals, namely generator-based spoofing, or by replaying the recorded authentic satellite signals, namely receiver-based spoofing or meaconing. Since the spoofing signals have similar temporal and spectral characteristics to authentic signals, it is more challenging to detect and mitigate such interference. In recent years, an increasing number of research groups have been involved in the study of spoofing countermeasures [11,12]. Most of them focus on spoofing detection based on a single antenna, such as amplitude discrimination [13], polarization discrimination [14], and the time-of-arrival (TOA) discrimination [15]. However, merely detecting the presence of a spoofing attack is not enough, and the ultimate goal of anti-spoofing is to eliminate spoofing signals and recover the positioning and timing capabilities of the victim receiver. The anti-spoofing techniques based on the antenna array, which is rising recently, not only can analyze the spatial signature of the received signals and identify spatially correlated spoofing signals, but also mitigate them by nulling technology [12]. These kinds of techniques can be implemented at the pre-despreading or post-despreading stage of a GNSS receiver. A pre-despreading method in [16] cross-correlated the baseband samples from different antennas in order to form a spatial correlation matrix and extracted the eigenvector corresponding to the maximum eigenvalue as the spoofing subspace. Then, projecting the array signal into its orthogonal subspace mitigated the spoofing signals. The basic idea is that all spoofing signals come from the same direction, the power density of which is higher than the other directions. Although this method has low complexity, it is difficult to determine the detection threshold because the navigation signals arriving at the receiver are generally below the noise level, whether it is an authentic signal or spoofing signal. In the post-despreading methods, the correlation and accumulation processes have been applied to each antenna sample [17,18]. Then, the directions-of-arrival (DOAs) of all the incoming navigation signals are estimated to distinguish between the spoofing and authentic signals. This method can not only ensure the gain of the authentic satellite signals through beamforming [19] while eliminating the spoofing signals, but also provide support for interference source positioning in some applications. Nonetheless, higher computational complexity makes it difficult to put into practice due to a large number of correlators that are needed for the receiver.

It is worth mentioning that there is a more complicated interference scenario where jamming and spoofing coexist. For example, in a confrontational environment, the jamming makes the target receiver loss-of-lock in a short time, and then the spoofing with higher power than the satellite signal leads the receiver to lock onto a false peak during reacquisition. Another possibility is to transmit high-power jamming signals and latent spoofing signals at the same time. Since most receivers on the market have strong capabilities of anti-jamming, this strategy can raise the probability of making the victim receiver fail in its positioning.

For such complex situations, the existing countermeasures are mostly a combination of adaptive spatial filtering based on array antennas and single antenna-based spoofing detection. Some schemes can suppress both jamming and spoofing by spatial processing. The authors in [20] introduced the subspace projection technique to eliminate jamming signals and exploit the despread-respread method to suppress spoofing interference. The despread-respread method [21], as a post-despreading method, requires repeated multi-peak acquisition processes for all pseudo-random noise (PRN) codes, thus increasing the computational burden of the receiver significantly. However, the acquisition threshold is difficult to determine in practice. If the threshold is too large, it can miss the possible false signal. If it is too small, it can be susceptible to multipath effects, resulting in a high false alarm rate. As for the pre-despreading methods, the authors in [22] employed the cross-spectral self-coherence restoral (cross-SCORE) algorithm to mitigate jamming and spoofing signals simultaneously. It presents a new idea that the navigation signal component can be enhanced in the cross-covariance matrix due to the self-coherence of the C/A code. However, the authors found in the simulation that this approach would fail when periodic jamming occurs, and the spoofing detection performance is sensitive to the location and length of the data block that is selected to estimate the cross-covariance matrix.

This paper aims to propose a novel GNSS interference suppression scheme using an antenna array that can detect and mitigate both jamming and spoofing signals before the despreading process of the receiver and reach a compromise between the computational cost and interference suppression capability. Since the two types of interference have entirely different signal characteristics, a two-stage structure was used to cope with them in turn. In the first stage, the spatial correlation matrix of the received signal is estimated. By performing the eigenvalue decomposition (EVD) on this matrix, the number of jamming signals and the jamming subspace can be easily determined because the jamming power is much higher than the noise level. Then, the array signal is projected into the jamming’s orthogonal subspace to eliminate the jamming signals. In the next stage, in order to deal with the spoofing signals with low power, the authors make full use of the cyclostationarity of GNSS signals to construct a cyclic correlation matrix, in which the noise component is significantly reduced or even removed. Thus, the signal subspace, which includes information about the power and DOAs of the GNSS signals, can be obtained before the despreading process. Then, a novel cyclic correlation eigenvalue test (CCET) algorithm is proposed to detect the presence of a spoofing attack, in which a test statistic is calculated based on the principal eigenvalues of the cyclic correlation matrix and then compared to a predefined threshold. The only assumption on this spoofing detection method is that all the spoofing signals are transmitted from a single-antenna source. Afterward, the cyclic music signal classification (Cyclic MUSIC) algorithm is employed to estimate the DOAs of all the navigation signals. Finally, subspace projection is again utilized to eliminate spoofing signals and meanwhile perform beamforming for each authentic satellite signal to overcome the power reduction caused by interference nulls.

The main contributions of this paper can be summarized as follows:

(1) A two-stage GNSS interference suppression scheme is proposed, in which the subspace projection instead of the conventional adaptive spatial filtering technique is adopted to remove jamming signals so that spoofing signals can be detected and mitigated by the spatial processing technology based on the array antenna.
(2) Due to all of the above, the operations are performed on the digitized baseband samples before the despreading process. The proposed technique does not introduce additional computational complexity to the GNSS receiver. Therefore, it is convenient to apply in real systems.
(3) Compared with other anti-spoofing methods implemented at the pre-despreading stage, such as the above-mentioned one [16], the proposed scheme not only suppresses jamming and spoofing effectively but also provides a higher gain in the directions of the desired satellite signal. Nonetheless, the estimated DOA of spoofing signals may be helpful for the interference source positioning in some applications.
(4) The proposed technique is effective only when the number of array elements is higher than the number of signals (include interference and satellite signals). Therefore, a suboptimal scheme is provided for the applications using small arrays, in which the maximum gain requirement for the authentic signals is relaxed to ensure that the jamming and spoofing signals are successfully eliminated.

The rest of this paper is organized as follows. In Section 2, the interference scenario is described and the received signal model is introduced. Then, the two-stage interference suppression scheme is presented in Section 3. In Section 4, the performance of the proposed spoofing detection is evaluated through theoretical analysis and simulation results. In Section 5, more simulation results are provided to validate the proposed scheme in different application scenarios. Section 6 concludes this paper.

## 2. Signal Model

**Figure 1:**
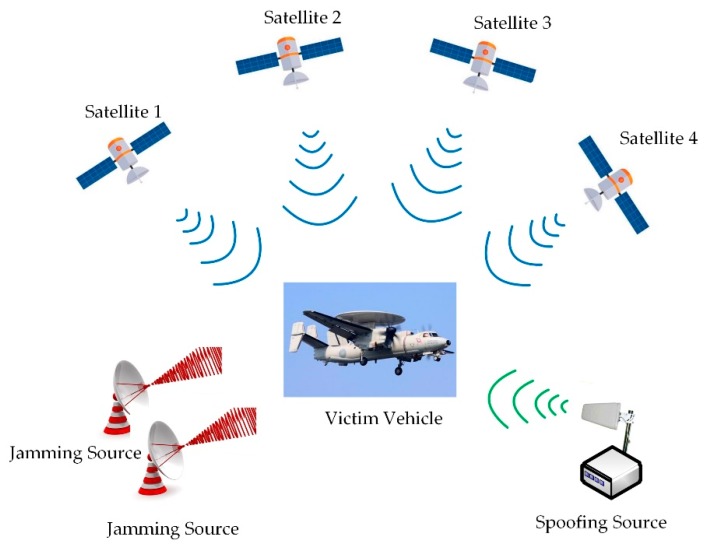
Illustration of an intentional attack on a global navigation satellite system (GNSS) receiver mounted on an aerial vehicle.

This paper focuses on the complicated interference scenario where both jamming and spoofing exist. As an example, Figure 1 illustrates an intentional attack on a GNSS receiver mounted on an aerial vehicle. Herein, there are likely one or several jamming sources emitting high-power radio frequency (RF) interference, while the spoofing source generally uses a single-antenna to transmit all the false signals, whether it is generator-based spoofing or receiver-based spoofing.

### 2.1. Received Array Signal

Without the loss of generality, it is assumed that $M^{A}$ authentic satellite signals, $M^{S}$ spoofing signals and $M^{J}$ jamming signals arrive at an *N*-element antenna array. Each element of the antenna array is connected to an RF front end and the resulting baseband sampled signals constitute the N×1 array signal vector as follows:

(1) $$x(nT_{s})=\sum_{m=1}^{M^{A}}a_{m}^{A}s_{m}^{A}(nT_{s})+\sum_{p=1}^{M^{S}}a_{p}^{S}s_{p}^{S}(nT_{s})+\sum_{q=1}^{M^{J}}a_{q}^{J}j_{q}(nT_{s})+n(nT_{s})$$

where $T_{s}$ is the sampling interval. Each row of $x(nT_{s})$ denotes the received signal by the corresponding array element and $n(nT_{s})$ is a complex additive white Gaussian noise vector. $j_{q}(nT_{s})\;(q=1,\cdots M^{J})$ represents the *j*th jamming signal; $s_{m}^{A}(nT_{s})\;(m=1,\cdots M^{A})$ denotes the *m*th authentic satellite signal, $s_{p}^{S}(nT_{s})\;(p=1,\cdots M^{S})$ means the *p*th spoofing signal and

(2) $$\begin{array}{l} s_{m}^{A}(nT_{s})=\sqrt{P_{m}^{A}}D_{m}^{A}(nT_{s}-\tau_{m}^{A})C_{m}^{A}(nT_{s}-\tau_{m}^{A})e^{j2\pi \left(f_{IF}+f_{m}^{A}\right)nT_{s}+j\phi_{m}^{A}}\\ s_{p}^{S}(nT_{s})=\sqrt{P_{p}^{S}}D_{p}^{S}(nT_{s}-\tau_{p}^{S})C_{p}^{S}(nT_{s}-\tau_{p}^{S})e^{j2\pi \left(f_{IF}+f_{p}^{S}\right)nT_{s}+j\phi_{p}^{S}} \end{array}$$

in which $f_{IF}$ is the intermediate frequency (IF), symbols P, τ, f, ϕ represent the power, code delay, Doppler frequency and phase of each signal, and the superscripts A, S refer to the authentic satellite and spoofing signal, respectively. $D(nT_{s})$ is the navigation data bit and $C(nT_{s})$ is the PRN sequence that identifies each satellite. Depending on the type of the spoofing attack, the number of spoofing PRNs can be the same or different from the authentic ones. The differences of the code delay and the Doppler frequency between the spoofing and authentic signals can be designed optionally. However, the power level of each spoofing signal should be comparable to that of its corresponding authentic one. 

**Figure 2:**
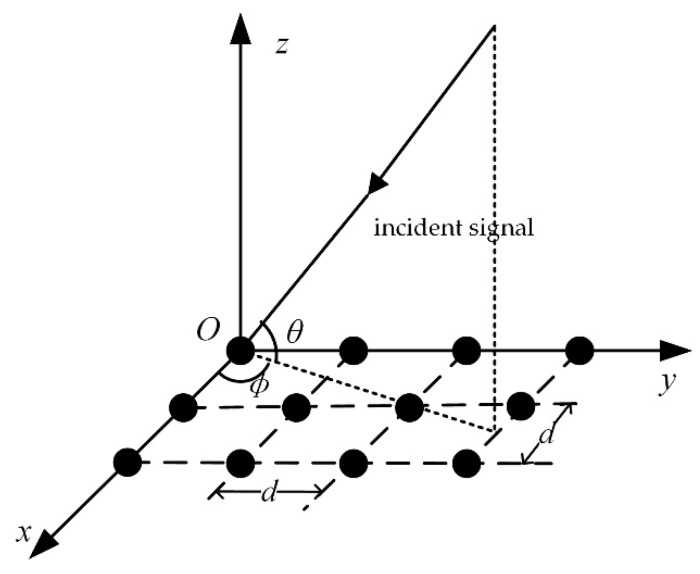
Local antenna coordinate system.

In Equation (1), the symbols $a_{m}^{A},\;a_{p}^{S},\;a_{q}^{J}$ denote the array steering vectors of the authentic satellite signals, the spoofing signals, and the jamming signals respectively. They describe the carrier phase differences of the received signals from the different antenna channels in specific directions [23]. Figure 2 shows the local antenna coordinate system, in which the x-axis and y-axis lie in the planar array and the z-axis points to the normal direction of the array, forming a right-hand coordinate system. Assume that the direction of the incoming signal is depicted by the angle pair γ=(θ,φ), with θ as the angle off the x−y plane and φ as the angle off the x-axis within the x−y plane. The incident direction vector is presented as:

(3) $$g(\gamma )=-{\left[\cos\theta \cos\varphi ,\cos\theta \sin\varphi ,\sin\theta \right]}^{T}$$

Ideally, the steering vector of this incoming signal can be expressed as follows:

(4) $$a(\gamma )={\left[e^{-j\frac{2\pi }{\lambda }p_{1}^{T}g(\gamma )},e^{-j\frac{2\pi }{\lambda }p_{2}^{T}g(\gamma )},\cdots ,e^{-j\frac{2\pi }{\lambda }p_{N}^{T}g(\gamma )}\right]}^{T}$$

where $p_{k}={\left[x_{k},y_{k},z_{k}\right]}^{T}\;(k=1,\cdots N)$ is the position vector of the *k*th element and λ denotes the wavelength of the incident signal. 

It can be seen that the signals incident on the array in the same direction have the same steering vector. Therefore, based on the assumption of single-antenna spoofing source, the received signal model in Equation (1) can be rewritten as:

(5) $$x(nT_{s})=\sum_{m=1}^{M^{A}}a_{m}^{A}s_{m}^{A}(nT_{s})+a^{S}\sum_{p=1}^{M^{S}}s_{p}^{S}(nT_{s})+\sum_{q=1}^{M^{J}}a_{q}^{J}j_{q}(nT_{s})+n(nT_{s})$$

in which $a^{S}$ is the same steering vector of all the spoofing signals.

### 2.2. Cyclostationarity of Global Positioning System (GPS) L1 Signals

It is well known that most GNSS systems employ PRN codes that are derived from linear shift-register sequences owing to their superior correlation properties. Considering the civilian GPS L1 signal, the signal $C(nT_{s})$ in Equation (2) is the periodic replication of a specific PRN code sequence of 1023 chips for each satellite [24]. Therefore, each GPS L1 signal exhibits a cyclostationarity at the code period $T_{C/A}=N_{c}T_{c}$, where $N_{c}=1023$ and $T_{c}=1/1.023\;\mathrm{MHz}$ is the chip period.

A signal is considered to be cyclostationary if its cyclic autocorrelation function (CAF), defined as:

(6) $$R_{ss}^{cc}\left(\tau \right)=E\left\{s(t)s^{*}(t-\tau )\right\}$$

is non-zero with some lag parameter τ [25]. Note that the CAF in this paper is the abbreviation of the cyclic autocorrelation function rather than the well-known cross-ambiguity function. Due to the periodicity of the PRN codes, the CAF of the individual GPS L1 signal is also periodic and is non-zero when and only when $\tau =lT_{C/A}\;\left(l=1,2,3,\cdots \right)$. Therefore, it can be considered as a cyclostationary signal.

It is worth mentioning that the spoofing detection and mitigation technique proposed in this paper is applicable to all the GNSS signals with periodic PRN codes. For convenience, the following section is described in the context of GPS L1 C/A signals.

## 3. Proposed Interference Suppression Scheme

**Figure 3:**
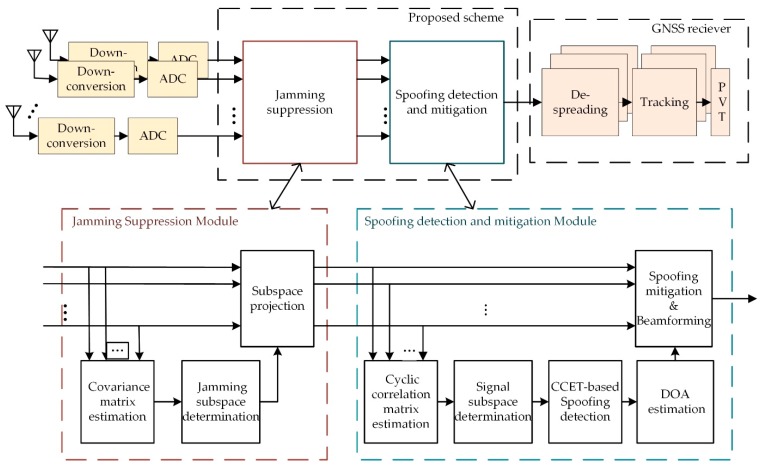
Block diagram of the proposed interference suppression scheme.

Figure 3 depicts the block diagram of the proposed interference suppression scheme. It is implemented in two stages, namely jamming suppression and spoofing detection and mitigation. In the jamming suppression module, the spatial covariance matrix of the received signal is firstly estimated. Then, the EVD of the covariance matrix is performed to determine the number of jamming signals and the eigenvectors of the jamming subspace. Finally, subspace projection is used to obtain a jamming-free signal vector. The second stage of this scheme is the detection and mitigation of a spoofing attack. It mainly contains five steps: (1) The cyclic correlation matrix estimation, (2) signal subspace determination, (3) spoofing detection based on the CCET algorithm, (4) DOA estimation and (5) subspace projection and beamforming. All the processing of the proposed scheme is performed on the raw digital baseband signal, and the resulting signals are passed to the despreading and tracking unit of the GNSS receiver for generating authentic PVT solutions. The details of these stages are provided in following subsections.

### 3.1. Jamming Suppression

Based on Equation (5), the covariance matrix of the received signal vector can be expressed as follows:

(7) $$\begin{array}{ll} R_{x} & =E\{x(nT_{s})x^{H}(nT_{s})\}\\ & =\sum_{q=1}^{M^{J}}R_{q}^{J}\left(nT_{s}\right)a_{q}^{J}{\left(a_{q}^{J}\right)}^{H}+\sum_{m=1}^{M^{A}}R_{m}^{A}\left(nT_{s}\right)a_{m}^{A}{\left(a_{m}^{A}\right)}^{H}+\left(\sum_{p=1}^{M^{S}}R_{p}^{S}\left(nT_{s}\right)\right)a^{S}{\left(a^{S}\right)}^{H}+\sigma_{n}^{2}I \end{array}$$

which is generally estimated by *K* samples in practice using the following formula:

(8) $${\hat{R}}_{x}=\frac{1}{K}\sum_{k=0}^{K-1}x(kT_{s})x^{H}(kT_{s})$$

In Equation (7),

(9) $$\begin{array}{l} R_{q}^{J}\left(nT_{s}\right)=E\left\{s_{q}^{J}(nT_{s})s_{q}^{J*}(nT_{s})\right\}=P_{q}^{J}\\ R_{m}^{A}\left(nT_{s}\right)=E\left\{s_{m}^{A}(nT_{s})s_{m}^{A*}(nT_{s})\right\}=P_{m}^{A}\\ R_{p}^{S}\left(nT_{s}\right)=E\left\{s_{p}^{S}(nT_{s})s_{p}^{S*}(nT_{s})\right\}=P_{p}^{S} \end{array}$$

are the values of the autocorrelation function of the *j*th jamming signal, the *m*th satellite signal and the *p*th spoofing signal, respectively, indicating the corresponding signal strength. In most situations, the power of satellite signal is approximately *20~30* dB lower than the noise and the spoofing signal is slightly higher than the authentic signal but still below the noise level, while jamming is usually much stronger than the noise. That is, the power of them satisfies:

(10) $$P_{q}^{J}\gg \sigma_{n}^{2}\gg P_{p}^{S}\geq P_{m}^{A}$$

where $q=1,\cdots ,M^{J},\;p=1,\cdots ,M^{S},\;m=1,\cdots ,M^{A}$. Hence, the covariance matrix can be approximated as the sum of the jamming covariance matrix and the noise covariance matrix as follows:

(11) $$\begin{array}{ll} {\hat{R}}_{x} & \approx {\hat{R}}_{J}+{\hat{R}}_{n}\\ & \approx \sum_{q=1}^{M^{J}}P_{q}^{J}a_{q}^{J}{\left(a_{q}^{J}\right)}^{H}+\sigma_{n}^{2}I \end{array}$$

In order to obtain the jamming subspace, the EVD of the covariance matrix and select corresponding eigenvectors of the $M^{J}$ largest eigenvalues can be performed. Assume that the EVD of ${\hat{R}}_{x}$ is given by

(12) $${\hat{R}}_{x}=\sum_{i=1}^{N}{\hat{\beta }}_{i}{\hat{e}}_{i}{\hat{e}}_{i}^{H}$$

where ${\hat{\beta }}_{1},{\hat{\beta }}_{2},\cdots ,{\hat{\beta }}_{N}$ are the eigenvalues in descending order and ${\hat{e}}_{1},{\hat{e}}_{2},\cdots ,{\hat{e}}_{N}$ are the normalized eigenvectors. Under the premise that the number of the jamming sources is unknown, we determine the number of the large eigenvalues are determined based on the following criterion [23]

(13) $$\{\begin{array}{ll} \frac{{\hat{\beta }}_{j}}{\sum_{i=1}^{N}{\hat{\beta }}_{i}}>T_{1}^{J} & \left(j=1,\cdots ,N\right)\\ \frac{{\hat{\beta }}_{j+1}}{{\hat{\beta }}_{j}}>T_{2}^{J} & \left(j=1,\cdots ,N-1\right) \end{array}$$

in which $T_{1}^{J},T_{2}^{J}$ are the test thresholds. The first metric is the ratio of the *j*th eigenvalue to the sum of all the eigenvalues, denoting the percent of the total power contained in ... If it exceeds the threshold, ${\hat{\beta }}_{j}$ is considered as a large eigenvalue. The second metric is the ratio of the (*j* + 1) th eigenvalue to the *j*th eigenvalue which has been declared to be large. If it is tiny, even approximately zero, then the eigenvalues ${\hat{\beta }}_{k}\left(k=j+1,\cdots ,N\right)$ correspond to the noise subspace.

Then, the number of large eigenvalues is regarded as the dimension of the jamming subspace and the first ${\hat{M}}^{J}$ eigenvectors construct the jamming subspace:

(14) $$\left\{ \begin{array}{ll} V_{J}=\left[{\hat{e}}_{1},{\hat{e}}_{2},\cdots ,{\hat{e}}_{M^{J}}\right] & {\hat{M}}^{J}\geq 1\\ V_{J}=0 & {\hat{M}}^{J}=0 \end{array}\right.$$

Accordingly, the orthogonal complement space to $V_{J}$ is defined as:

(15) $$V_{⊥}^{J}=I-V_{J}{\left(V_{J}^{H}V_{J}\right)}^{-1}V_{J}^{H}$$

which meets:

(16) $$V_{⊥}^{J}a_{q}^{J}\approx 0\;(q=1,\cdots ,M^{J})$$

Therefore, projecting the received signal in this orthogonal complement space can suppress jamming and the output signal vector is given by:

(17) $$\begin{array}{ll} y(nT_{s}) & =V_{⊥}^{J}x(nT_{s})\\ & =\sum_{m=1}^{M^{A}}b_{m}^{A}s_{m}^{A}(nT_{s})+b^{S}\sum_{p=1}^{M^{S}}s_{p}^{S}(nT_{s})+\tilde{n}(nT_{s}) \end{array}$$

where $b_{m}^{A}=V_{⊥}^{J}a_{m}^{A},\;b^{S}=V_{⊥}^{J}a^{S}$ denote the new steering vectors of the authentic satellite signal and spoofing signals respectively, and $\tilde{n}(nT_{s})=V_{⊥}^{J}n(nT_{s})$ is the new noise vector. Note that the new steering vector is still an *N*-dimensional column vector but the subspace projection reduces its spatial degree of freedom (DOF) to be $\left(N-{\hat{M}}^{J}\right)$.

### 3.2. Spoofing Detection and Mitigation

In the output of the jamming suppression module, the power of the spoofing signals and satellite signals are still below the noise level. In order to cope with the spoofing signals before the despreading operation of the receiver, the particular characteristics of the GPS signals have to be excavated sufficiently. As mentioned in Section 2, each GPS L1 signal is a cyclostationary sequence that has a periodic cyclic autocorrelation function, and the value of the CAF is non-zero when and only when the lag parameter is $\tau =lT_{C/A}\;\left(l=1,2,3,\cdots \right)$. Therefore, in order to concentrate on the signal components in the spatial correlation matrix and remove the noise component, the cyclic correlation matrix should be calculated, which is defined as the cross-correlation of the received signal vector and its delayed version as follows:

(18) $$\begin{array}{ll} R_{y}^{c} & =E\left\{y(nT_{s})y^{H}(nT_{s}-T_{c/a})\right\}\\ & =\sum_{m=1}^{M^{A}}b_{m}^{A}{\left(b_{m}^{A}\right)}^{H}R_{m^{A}m^{A}}^{cc}\left(T_{C/A}\right)+b^{S}{\left(b^{S}\right)}^{H}\sum_{p=1}^{M^{S}}R_{p^{S}p^{S}}^{cc}\left(T_{C/A}\right)+\sum_{i=1}^{M^{A}}b_{i}^{A}{\left(b^{S}\right)}^{H}R_{i^{S}i^{A}}^{cc}\left(T_{C/A}\right) \end{array}$$

where $T_{c/a}$ is the C/A code period, which is almost equal to 1 ms for each GPS L1 signal without considering the influence of code Doppler.

$R_{m^{A}m^{A}}^{cc}\left(T_{C/A}\right)$ denotes the value of the CAF of the *m*th $\left(m=1,\cdots M^{A}\right)$ satellite signal at $T_{c/a}$. Neglecting the navigation data bits, it can be expressed as:

(19) $$\begin{array}{ll} R_{m^{A}m^{A}}^{cc}\left(T_{C/A}\right) & =E\left\{s_{m}^{A}(nT_{s})s_{m}^{A*}(nT_{s}-T_{C/A})\right\}\\ & =P_{m}^{A}e^{j2\pi \left(f_{IF}+f_{m}^{A}\right)T_{c/a}} \end{array}$$

Similarly,

(20) $$\begin{array}{ll} R_{p^{S}p^{S}}^{cc}\left(T_{C/A}\right) & =E\left\{s_{p}^{S}(nT_{s})s_{p}^{S*}(nT_{s}-T_{C/A})\right\}\\ & =P_{p}^{S}e^{j2\pi \left(f_{IF}+f_{p}^{S}\right)T_{c/a}} \end{array}$$

is the value of CAF of the *p*th $\left(p=1,\cdots M^{S}\right)$ spoofing signal at $T_{c/a}$.

Knowing that the Doppler frequency shifts for the baseband GPS signals are generally between −5 kHz and +5 kHz, the following approximation can be made:

(21) $$e^{j2\pi \left(f_{IF}+f_{m}^{A}\right)T_{c/a}}\approx e^{j2\pi \left(f_{IF}+f_{p}^{S}\right)T_{c/a}}\approx e^{j2\pi f_{IF}T_{c/a}}≜C_{IF}$$

where $C_{IF}$ is defined as a complex constant, the norm of which is 1. Accordingly, Equation (19), (20) can be simplified to:

(22) $$\begin{array}{c} R_{m^{A}m^{A}}^{cc}\left(T_{C/A}\right)\approx C_{IF}P_{m}^{A}\\ R_{p^{S}p^{S}}^{cc}\left(T_{C/A}\right)\approx C_{IF}P_{p}^{S} \end{array}$$

The last term of Equation (18) is the cross correlation between the satellite and spoofing signals with the same PRN code $C_{i}^{A}(t)=C_{i}^{S}(t)=C_{i}(t)$, which can be expressed as:

(23) $$\begin{array}{ll} R_{i^{S}i^{A}}^{cc}\left(T_{C/A}\right) & =E\left\{s_{i}^{A}(t)s_{i}^{S*}(nT_{s}-T_{C/A})\right\}\\ & \approx \sqrt{P_{i}^{A}}\sqrt{P_{i}^{S}}E\left\{C_{i}(nT_{s}-\tau_{i}^{A})C_{i}(nT_{s}-\tau_{i}^{S}-T_{C/A})\right\}e^{j2\pi \left(f_{i}^{A}-f_{i}^{A}\right)T_{C/A}} \end{array}$$

As can be seen, its value depends on the code delay difference and the Doppler frequency difference between the satellite signal and its counterfeit signal. In general, the Doppler frequency difference between the spoofing signal and the corresponding satellite signal is a few Hz, it can be written $\left(f_{i}^{A}-f_{i}^{A}\right)T_{C/A}\ll 1$ and the phase rotation can be neglected. Equation (23) can be denoted as:

(24) $$R_{i^{A}i^{S}}^{cc}\left(T_{C/A}\right)\approx \rho_{ii}^{AS}\sqrt{P_{i}^{A}}\sqrt{P_{i}^{S}}$$

where $\rho_{ii}^{AS}\left(0\leq \rho_{ii}^{AS}\leq 1\right)$ is the correlation result of $C_{i}(nT_{s}-\tau_{i}^{A})$ and $C_{i}(nT_{s}-\tau_{i}^{S}-T_{C/A})$. In general, the spoofing signals are designed to be more than one chip delay or advance relative to the authentic signals, and $C_{i}(nT_{s}-\tau_{i}^{A})$ and $C_{i}(nT_{s}-\tau_{i}^{S}-T_{C/A})$ can be considered to be uncorrelated, that is, $R_{i^{S}i^{A}}^{cc}\left(T_{C/A}\right)=0$ [24]. However, in some complicated scenarios, the code phase differences may be within one chip, which makes it difficult to distinguish between spoofing and authentic signals by the time-domain methods. 

In Equation (18), the cross-correlation matrix of each GPS signal and noise vector and the cross-correlation matrix of the noise vector and its delayed version have been eliminated because the noise is assumed to be Gaussian.

Without the loss of generality, the authors regard spoofing detection as a binary statistical hypothesis testing problem with $H_{0}$ denoting the null hypothesis that there is no spoofing attack and with $H_{1}$ denoting the null hypothesis that spoofing attack is present. Equation (18) is reformulated as:

(25) $$R_{y}^{c}=\{\begin{array}{ll} \sum_{m=1}^{M^{A}}\left(C_{IF}P_{m}^{A}\right)b_{m}^{A}{\left(b_{m}^{A}\right)}^{H} & H_{0}\\ \sum_{m=1}^{M^{A}}\left(C_{IF}P_{m}^{A}\right)b_{m}^{A}{\left(b_{m}^{A}\right)}^{H}+\left(C_{IF}\sum_{p=1}^{M^{S}}P_{p}^{S}\right)b^{S}{\left(b^{S}\right)}^{H}+\sum_{i=1}^{M^{A}}\left(\rho_{ii}^{AS}\sqrt{P_{i}^{A}}\sqrt{P_{i}^{S}}\right)b_{i}^{A}{\left(b^{S}\right)}^{H} & H_{1} \end{array}$$

As can be observed, when there is no spoofing attack, the rank of the cyclic correlation matrix is equal to the number of satellite signals, i.e., $rank\left(R_{y}^{c}\right)=M^{A}$. By computing the EVD, $M^{A}$ non-zero eigenvalues and $\left(N-M^{A}\right)$ zero eigenvalues can be obtained. The first $M^{A}$ eigenvectors is a set of the orthogonal basis of the signal space spanned by the signal steering vectors $\left\{b_{1}^{A},b_{2}^{A},\cdots ,b_{M^{A}}^{A}\right\}$, and the remaining eigenvectors corresponding to the zero eigenvalues form the null space. Therefore, the DOAs of signals can be estimated by the Cyclic MUSIC algorithm [26]. The difference between this algorithm and the traditional MUSIC algorithm is that it uses the cyclic correlation matrix instead of the covariance matrix.

Whereas if a spoofing attack is present, $H_{1}$ consists of two cases. (i) When each spoofing signal is off the authentic counterpart by more than one chip in time, the last term in Equation (25) is negligible. The rank of the cyclic correlation matrix becomes $\left(M^{A}+1\right)$ and the signal subspace contains the steering vectors of satellite signals and spoofing signals as $\left\{b^{S},b_{1}^{A},b_{2}^{A},\cdots ,b_{M^{A}}^{A}\right\}$. Due to the power of all the spoofing signals are combined in a specific steering vector, there should be a significantly larger eigenvalue in the $\left(M^{A}+1\right)$ principal eigenvalues. The maximum peak location of the spatial power spectrum estimated by the Cyclic MUSIC algorithm indicates the DOA of the spoofing signals. (ii) When the code phase difference is within one chip, the performance of the Cyclic MUSIC algorithm depends on the correlation between the spoofing and authentic signals. If the spoofing is close to the authentic signal, the high correlation may cause rank deficiency of the signal subspace. In the follow-up simulations, it has been found that when the offset between spoofing and authentic signals is less than the 0.5 chips, the DOA of these signals cannot be estimated accurately. On the other hand, if the offset is more than the 0.5 chips, the correlation between the spoofing and authentic signals is not sufficient to make their DOA indistinguishable. In either case, because each pair of the correlated signals contains a spoofing signal, a significant component can still appear in the principal eigenvalues. The victim can detect the unusual eigenvalue and issue the spoofing alarm.

Therefore, in this section, the focus is on the case of weak correlation and a cyclic correlation eigenvalues test (CCET) algorithm is proposed to detect the presence of a spoofing attack and make the full use of the DOA estimation results to mitigate spoofing signals by subspace projection and enhance the authentic satellite signals through beamforming. The following subsections present the specific steps of the proposed technique.

#### 3.2.1. Cyclic Correlation Matrix Estimation

In practice, the cyclic correlation matrix can not be obtained accurately and has to be estimated by finite samples as follows:

(26) $${\tilde{R}}_{y}^{c}=\frac{1}{K}Y_{K}{\left(Y_{K}^{D}\right)}^{H}$$

where:

$$\begin{array}{l} Y_{K}=\left[y(k),y(k-1),\cdots ,y(k-K+1)\right]\\ Y_{K}^{D}=\left[y(k-D),y(k-D-1),\cdots ,y(k-D-K+1)\right] \end{array}$$

are the N×K data matrix and respective delayed matrix, K is the length of the data block and D is the number of samples in one code period (1 ms). 

**Figure 4:**
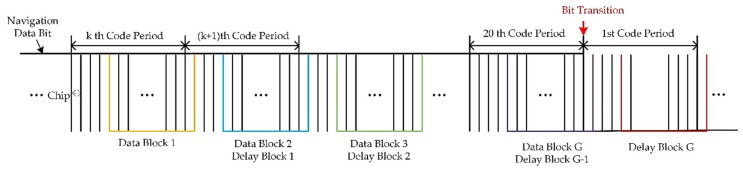
GPS L1 C/A signal structure and the data block for the cyclic correlation matrix estimation.

This is the most direct way of estimating the cyclic correlation matrix, which is the most employed in the literature [22]. However, the authors found through the experiments that it might yield poor estimation performance when applied to a real system. This is because the data samples used for cyclic correlation matrix estimation are selected randomly and the length of the data block is limited. Take one of the satellite or spoofing signals as an example, and GPS L1 C/A signal structure is shown in Figure 4. Several pairs of data blocks are marked in the figure, and it is noted that the Data Block G (purple line) is split between two adjacent symbols with opposite signs. If this data block is used for estimating ${\tilde{R}}_{y}^{c}$, the correlation result of this signal can be weakened. 

This paper solves this problem by using multiple data blocks to get many more correlation matrixes. As shown in Figure 4, G (1≤G<20) data blocks are selected and the averaging correlation matrix can be expressed as:

(27) $${\hat{R}}_{y}^{c}=\frac{1}{G}\sum_{g=1}^{G}{\left({\hat{R}}_{y}^{c}\right)}^{g}=\frac{1}{G}\sum_{g=1}^{G}\frac{1}{K}Y_{K}^{g}{\left(Y_{K}^{gD}\right)}^{H}$$

where

$$\begin{array}{l} Y_{K}^{g}=\left[y(k-gD),y(k-1-gD),\cdots ,y(k-K+1-gD)\right]\\ Y_{K}^{gD}=\left[y(k-(g+1)D),y(k-1-(g+1)D),\cdots ,y(k-K+1-(g+1)D)\right] \end{array}$$

are the *g*th data block and the *g*th delayed data block. For each GPS L1 C/A signal, at most, one pair of these data blocks may suffer from symbol transition, while the others belong to one symbol. 

However, it is worth noting that Equation (27) is generally not Hermitian, resulting in the EVD cannot be performed. It needs to be turned into a conjugate symmetry matrix by:

(28) $${\left({\hat{R}}_{y}^{c}\right)}^{g*}=\frac{1}{2}\left(\frac{1}{K}Y_{K}^{g}{\left(Y_{K}^{gD}\right)}^{H}+\frac{1}{K}Y_{K}^{gD}{\left(Y_{K}^{g}\right)}^{H}\right)$$

which has been proved to have similar statistical properties with ${\left({\hat{R}}_{y}^{c}\right)}^{g}$ in [27].

Summing up the above, the estimation of the cyclic correlation matrix is given by:

(29) $${\hat{R}}_{y}^{c}=\frac{1}{G}\sum_{g=1}^{G}\frac{1}{2K}\left(Y_{K}^{g}{\left(Y_{K}^{gD}\right)}^{H}+Y_{K}^{gD}{\left(Y_{K}^{g}\right)}^{H}\right)$$

#### 3.2.2. Signal Subspace Determination

Due to the cyclic correlation matrix is estimated by finite samples, in practice, there are no zero eigenvalues but only small eigenvalues. The dimension of the signal subspace d based on the minimum description length (MDL) criterion [28] needs to be estimated before spoofing detection and DOA estimation. Denoting the EVD of ${\hat{R}}_{y}^{c}$ as follows:

(30) $${\hat{R}}_{y}^{c}=\sum_{i=1}^{N}{\hat{\lambda }}_{i}{\hat{u}}_{i}{\hat{u}}_{i}^{H}$$

where ${\hat{\lambda }}_{1},{\hat{\lambda }}_{2},\cdots ,{\hat{\lambda }}_{N}$ are the eigenvalues in descending order and ${\hat{u}}_{1},{\hat{u}}_{2},\cdots ,{\hat{u}}_{N}$ are the normalized eigenvectors. The MDL estimator of the signal subspace dimension is given by:

(31) $$\hat{d}=\arg\underset{d=0,\cdots N-{\hat{M}}^{J}}{\min}\left\{L_{d}\left(d\right)+\frac{1}{2}\left[d\left(N-{\hat{M}}^{J}-d\right)+1\right]\ln\left(GK\right)\right\}$$

where

(32) $$L_{d}\left(d\right)=GK\left(N-{\hat{M}}^{J}-d\right)\ln\left\{\frac{\frac{1}{N-{\hat{M}}^{J}-d}\sum_{k=d+1}^{N-{\hat{M}}^{J}}{\hat{\lambda }}_{k}}{{\left(\prod_{k=d+1}^{N}{\hat{\lambda }}_{k}\right)}^{\frac{1}{N-{\hat{M}}^{J}-d}}}\right\}$$

is the log-likelihood function and *GK* is the number of all samples for estimating ${\hat{R}}_{y}^{c}$. It is noticeable that the used set of eigenvalues is $\left\{{\hat{\lambda }}_{1},{\hat{\lambda }}_{2},\cdots ,{\hat{\lambda }}_{N-{\hat{M}}^{J}}\right\}$ instead of $\{{\hat{\lambda }}_{1},{\hat{\lambda }}_{2},\cdots ,{\hat{\lambda }}_{N}\text{\}}$. This is because the jamming suppression module has reduced the rank of the correlation matrix through subspace projection, resulting in the latter ${\hat{M}}^{J}$ eigenvalues being negligible.

Then, the first $\hat{d}$ eigenvectors construct the signal subspace:

(33) $${\hat{U}}_{S}=\left[\begin{array}{llll} {\hat{u}}_{1} & {\hat{u}}_{2} & \cdots & {\hat{u}}_{\hat{d}} \end{array}\right]$$

and the remaining $\left(N-\hat{d}\right)$ eigenvectors form the null space:

(34) $${\hat{U}}_{N}=\left[\begin{array}{llll} {\hat{u}}_{\hat{d}+1} & {\hat{u}}_{\hat{d}+2} & \cdots & {\hat{u}}_{N} \end{array}\right]$$

#### 3.2.3. Spoofing Detection Based on the CCET Algorithm

This subsection describes the proposed spoofing detection method based on the distribution of the principal eigenvalues of the cyclic correlation matrix, which is referred to as the CCET algorithm.

**Figure 5:**
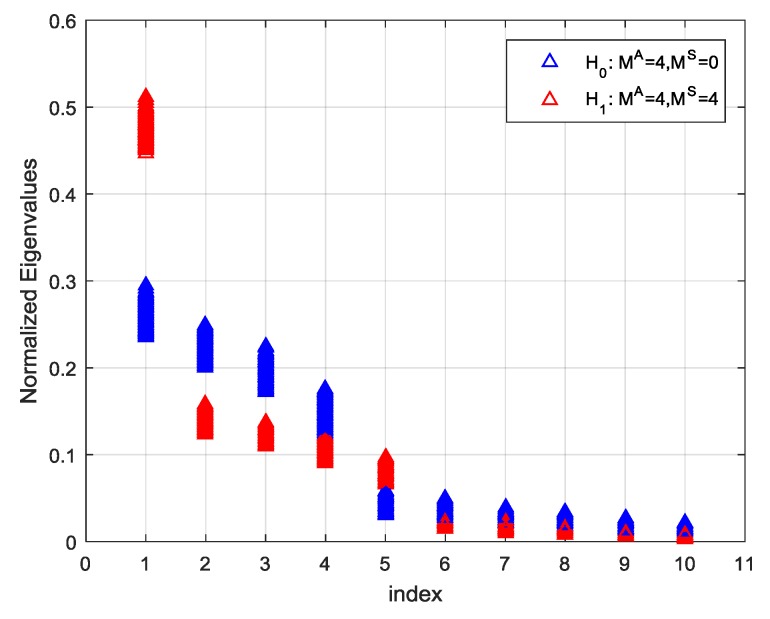
The distribution of the eigenvalues of the cyclic correlation matrix under $H_{0}$ and $H_{1}$.

As mentioned before, there is a significantly larger eigenvalue in the principal eigenvalues $\left\{{\hat{\lambda }}_{1},{\hat{\lambda }}_{2},\cdots ,{\hat{\lambda }}_{\hat{d}}\right\}$ if the spoofing attack exists. To clarify this point further, two groups of Monte-Carlo simulations were carried out with a 10-element uniform linear array (ULA) under $H_{0}$ and $H_{1}$. The four satellite signals were considered and the power of each signal was assumed to be –157 dBW. Under $H_{1}$, four spoofing signals with the same PRNs as the satellite signals have been assumed and the power of them was also −157 dBW. The code delay and Doppler frequency of each spoofing signals were randomly chosen but not equal to those of authentic signals. The power of additive White Gaussian noise was assumed to be −130 dBW. The run was repeated 1000 times. The normalized eigenvalues under $H_{0}$ and $H_{1}$ arranged in descending order, are shown in Figure 5.

As can be seen from the figure, the first four eigenvalues distribute approximately along a straight line concerning their indexes under $H_{0}$. In the other case, when several spoofing signals from one specific direction present, their power is superimposed to produce a significantly large eigenvalue, which is no longer consistent with the straight line formed by other ones. If a straight line is found to fit the points of ${\hat{\lambda }}_{i}\;(i=1,\cdots ,\hat{d})$ in a least-squares sense, the quality of the obtained solution can be assessed using the sum of squares of errors (SSE), which is defined by:

(35) $$SSE=\sum_{i=1}^{\hat{d}}{\left({\hat{\lambda }}_{i}-{\tilde{\lambda }}_{i}\right)}^{2}$$

where ${\tilde{\lambda }}_{i}$ is the points on the straight line corresponding to ${\hat{\lambda }}_{i}$.

Under $H_{0}$, the SSE metric follows a non-central chi-squared ($\chi^{2}$) distribution with $\hat{d}$ degrees of freedom and non-central parameter $\sigma_{0}$, which depends on the variance of the satellite signal power. When the power of all the satellite signals are equal, the residuals of the least- squares solution are unbiased and the SSE metric follows a central $\chi^{2}$ distribution [29]. Under $H_{1}$, the SSE metric follows a non-central $\chi^{2}$ distribution with the same degrees of freedom $\hat{d}$, but a bigger non-zero parameter $\sigma_{1}$ owing to the largest eigenvalue. Therefore, it is well-reasoned to take the SSE metric of linear fitting as the test statistic of spoofing detection, which follows:

(36) $$T_{sse}∼\{\begin{array}{l} \chi^{2}\left(\hat{d},\sigma_{0}\right)\\ \chi^{2}\left(\hat{d},\sigma_{1}\right) \end{array}\;\begin{array}{l} under\;H_{0}\\ under\;H_{1} \end{array}$$

Then the decision rule can be expressed as:

(37) $$T_{sse}\overset{H_{0}}{\underset{H_{1}}{\begin{array}{c} <\\ > \end{array}}}\eta$$

where η>0 is a threshold chosen to achieve an expected detection performance.

The false alarm probability $P_{FA}$ and the detection probability $P_{D}$ are vital parameters used to evaluate the performance of detection algorithms. The detection probability is the probability of being under $H_{1}$ and accurately detecting the spoofing attack. The false alarm probability is the probability of being under $H_{0}$ but mistakenly detecting a spoofing. That is,

(38) $$\begin{array}{l} P_{D}≜\mathrm{Pr}\left(T>\eta |H_{1}\right)\\ P_{FA}≜\mathrm{Pr}\left(T>\eta |H_{0}\right) \end{array}$$

An optimal threshold is required to improve the detection probability and reduce the false alarm probability as much as possible. In practical applications, the receiver may be up against different spoofing scenarios where the number and power of spoofing signals are unknown and the incoming direction is randomly varied. It is difficult to predict the probability distribution function (PDF) of the test statistic under $H_{1}$. Nevertheless, when the $H_{0}$ hypothesis is true, the PDF of the test statistic in different scenarios can be obtained where the number and power level of satellite signals are varied but known. Once the PDF under $H_{0}$ is determined, given a desired false alarm probability $P_{FA}$, the detection threshold η can be calculated by satisfying:

(39) $$\int_{\eta }^{\infty }f_{\chi^{2}}\left(x,d,\sigma_{0}\right)dx=P_{FA}$$

where $f_{\chi^{2}}\left(\cdot ,d,\sigma_{0}\right)$ is the PDF of a $\chi^{2}$ random variable with degree-of-freedom d and non- central parameter $\sigma_{0}$.

#### 3.2.4. DOA Estimation and Spoofing Mitigation

After the spoofing detection unit, the Cyclic MUSIC algorithm is adopted to estimate the DOAs of the navigation signals. Its basic idea is to estimate the spatial power spectrum by the signal subspace ${\hat{U}}_{S}$ obtained from the cyclic correlation matrix as follows:

(40) $$Q(\gamma )=\frac{1}{v(\gamma )\left(I-{\hat{U}}_{S}{\hat{U}}_{S}^{H}\right)v^{H}(\gamma )}$$

in which $v(\gamma )=P_{⊥}^{J}a(\gamma )$ is the steering vector of an incoming signal from γ=(θ,φ), and then search for its $\hat{d}$ largest peaks. In the spatial power spectrum, the location of the *i*th peak ${\hat{\gamma }}_{i}=({\hat{\theta }}_{i},{\hat{\varphi }}_{i})$ denotes the DOA of the *i*th signal and the value of the peak indicates the power density in that direction.

Depending on the result of spoofing detection, the subsequent process is distinct in the following two cases:

• Assume that $H_{1}$ is true

The location of the largest peak ${\hat{\gamma }}_{1}=({\hat{\theta }}_{1},{\hat{\varphi }}_{1})$ denotes the DOA of the spoofing signals and the spoofing steering vector can be estimated as follows:

(41) $${\hat{b}}^{S}=P_{⊥}^{J}a({\hat{\gamma }}_{1})$$

In the same way, the steering vectors of $\left(\hat{d}-1\right)$ authentic satellite signals are obtained by:

(42) $${\hat{b}}_{i}^{A}=P_{⊥}^{J}a({\hat{\gamma }}_{i+1})\;(i=1,\cdots ,\hat{d}-1)$$

Similar to the subspace projection method in Section 3.1, spoofing interference can be eliminated by projecting the array signal vector onto the null space of the spoofing subspace. The projection matrix is calculated by:

(43) $$P_{⊥}^{S}=I-\frac{{\hat{b}}^{S}{\left({\hat{b}}^{S}\right)}^{H}}{{\left({\hat{b}}^{S}\right)}^{H}{\hat{b}}^{S}}$$

Furthermore, to reduce unavoidable attenuation on the array pattern in the directions of authentic satellite signals due to jamming and spoofing nulls, the power of each authentic signal is maximized individually by beamforming. The array weight vector for the *i*th authentic satellite signals can be represented by:

(44) $$w_{i}^{H}={\left({\hat{b}}_{i}^{A}\right)}^{H}P_{⊥}^{S}$$

and the final output of the *i*th signal channel is given by:

(45) $$z_{i}\left(nT_{s}\right)=w_{i}^{H}y\left(nT_{s}\right)$$

• Assume that $H_{0}$ is true

The locations of the $\hat{d}$ peak indicate the DOAs of the authentic satellite signals and the corresponding estimated value of the steering vectors is expressed as follows:

(46) $${\hat{b}}_{i}^{A}=P_{⊥}^{J}a({\hat{\gamma }}_{i})\;(i=1,\cdots ,\hat{d})$$

The spoofing mitigation is no longer required in this case and the array weight vector in Equation (44) becomes:

(47) $$w_{i}^{H}={\left({\hat{b}}_{i}^{A}\right)}^{H}$$

### 3.3. Overall Interference Suppression Scheme

To summarize the proposed multiple interference suppression scheme, all the steps are listed in Algorithm 1. 

**Algorithm 1** Multiple Interference Suppression Scheme
**Jamming Suppression**

**Input:**
$x(nT_{s})$

(1)Estimate the spatial covariance matrix ${\hat{R}}_{x}=\frac{1}{K}\sum_{k=0}^{K-1}x(kT_{s})x^{H}(kT_{s})$.(2)Compute the EVD of ${\hat{R}}_{x}$, and obtain the jamming subspace $V_{J}$ and its orthogonal subspace $P_{⊥}^{J}$.(3)Project the received signal onto the jamming-free subspace $y(nT_{s})=P_{⊥}^{J}x(nT_{s})$.

**Output:**
$y(nT_{s}),P_{⊥}^{J},M^{J}$

**Spoofing Detection and Mitigation**

**Input:**
$y(nT_{s}),P_{⊥}^{J},M^{J}$

(1)Estimate the cyclic correlation matrix by Equation (28).(2)Compute the EVD of ${\hat{R}}_{y}^{c}$ and obtain the eigenvalues and eigenvectors of the signal subspace ${\hat{\lambda }}_{i},{\hat{u}}_{i}\;\left(i=1,\cdots ,\hat{d}\right)$.(3)Compute the test statistic $T_{sse}$ based on the CCET algorithm.(4)Decision. If $T_{sse}>\eta$, then the spoofing signals exist; otherwise, there is no spoofing signal.(5)Estimate the spoofing steering vector ${\hat{b}}^{S}$ and the authentic steering vectors ${\hat{b}}_{i}^{A}(i=1,\cdots ,\hat{d}-1)$ by the Cyclic MUSIC algorithm.(6)Compute the array weight vector for each satellite signal $w_{i}^{H}={\left({\hat{b}}_{i}^{A}\right)}^{H}P_{⊥}^{S}$ (under $H_{1}$) or $w_{i}^{H}={\left({\hat{b}}_{i}^{A}\right)}^{H}$ (under $H_{0}$)(7)Obtain the output signal $z_{i}\left(nT_{s}\right)=w_{i}^{H}y\left(nT_{s}\right)$

**Output:**
$z_{i}\left(nT_{s}\right)$


### 3.4. Countermeasure in the Case of Small Arrays

Notably, the above method is proposed under the premise that the number of array elements is higher than the number of signals (include interference and satellite signals). That is to say, after jamming the suppressing module, the remaining degree of freedom is still higher than the number of other signals so that the DOAs of the satellite signals and the spoofing signals can be obtained. However, in some small or agile applications, it may be not possible to install a large enough array. Under the circumstances, the requirement for the gain of the authentic signal must be relaxed to ensure that the spoofing signals are successfully eliminated.

When the EVD of ${\hat{R}}_{y}^{c}$ contains $\hat{d}=N-M^{J}$ non-zero eigenvalues, it denotes the inability to obtain the number of the signal sources and estimate their directions accurately. In this case, only spoofing signals can be detected by observing whether there is a relatively large eigenvalue. Similar to jamming detection, the authors predicate the existence of the spoofing attack if the largest eigenvalue satisfies:

(48) $$\{\begin{array}{ll} \frac{{\hat{\lambda }}_{1}}{{\hat{\lambda }}_{2}}>T_{1}^{S} & \left(a\right)\\ \frac{{\hat{\lambda }}_{1}}{\sum_{i=1}^{N-M^{J}}{\hat{\lambda }}_{i}}>T_{2}^{S} & \left(b\right) \end{array}$$

where $T_{1}^{S},T_{2}^{S}$ are the test threshholds. It is worth mentioning that the spoofing detection performance of this method is superior to that of the traditional pre-despreading technique. As the noise component has been greatly attenuated by the cyclic correlation process, the eigenvalues of ${\hat{R}}_{y}^{c}$ directly reflect the percent of the signal power in a specific direction in the total power.

Then project the array signal vector onto the null space of the spoofing subspace, and the final output is given by:

(49) $$z\left(t\right)=w_{S}^{H}P_{⊥}^{S}y\left(t\right)$$

where $P_{⊥}^{S}=I-{\hat{u}}_{1}{\hat{u}}_{1}^{H}{}_{1}$ is the projection matrix for spoofing mitigation and $w_{S}={\left[1,0,\cdots ,0\right]}^{H}$ denotes the weight vector resulting in the quiescent beam pattern [22], with the value of *1* corresponding to the reference element.

## 4. Performance Evaluation of the Spoofing Detection Method

In the proposed scheme, the jamming suppression module is simple in principle and significantly effective, while the spoofing detection and mitigation module is implemented in multiple steps and each step may involve errors when applied to real systems. In this section, both theoretical analysis and simulation results are presented to evaluate the performance of the proposed spoofing detection method.

The common simulation parameters are given as follows. A 10-element ULA was employed and the spacing between adjacent elements was half signal wavelength. The authentic and spoofing signals were generated with a Matlab-based GPS L1 C/A signal generator and they were sampled at a rate of 5 MHz. The additive Gaussian noise on each antenna was assumed to be white with spectral density N_0_ = −204 dBW/Hz. The power of authentic and spoofing signals varied based on the simulation scenarios.

### 4.1. Finite-Sample Effect on the Cyclic Correlation Matrix Estimation

In Section 3.2.2, the authors explained that the estimation performance of the cyclic correlation matrix can be improved by using multiple pairs of data blocks. In essence, it can be proved [30] that ${\hat{R}}_{y}^{c}$ in Equation (29) is an asymptotically unbiased estimator of $R_{y}^{c}$ and:

(50) $$E\left\{{\hat{R}}_{y}^{c}\right\}=\left(1-\rho \right)R_{y}^{c}$$

(51) $$E\left\{{\hat{R}}_{y}^{c}{\hat{R}}_{y}^{c}{}^{H}\right\}=\left(1+\left(\frac{2}{G}-2\right)\rho \right)\left(\frac{N\left(N+{\tilde{\sigma }}^{2}\right)}{K}R_{y}^{c}+\frac{N\left(1+{\tilde{\sigma }}^{2}\right)}{K}I\right)$$

On the right-hand side of Equation (51) is the expected cyclic correlation matrix multiplied by a constant and $\rho =T_{code}/T_{nav}$, in which $T_{code}$ and $T_{nav}$ denote the periods of the PRN code and the navigation data bit. For GPS L1 C/A signal, $T_{nav}=20T_{code}$. This attenuation coefficient (1−ρ) is due to the term cancellation when one of the *G* (1≤G<20) data blocks split between the two adjacent navigation symbols with opposite signs. 

Assume that:

(52) $${\hat{R}}_{y}^{c}=\left(1-\rho \right)R_{y}^{c}+N$$

N is a zero-mean error matrix, the variance of which can be expressed as:

(53) $$\mathrm{var}\left\{N\right\}=\left(1+\left(\frac{2}{G}-2\right)\rho \right)\left(\frac{N\left(N+{\tilde{\sigma }}^{2}\right)}{K}R_{y}^{c}+\frac{N\left(1+{\tilde{\sigma }}^{2}\right)}{K}I\right)-{\left(1-\rho \right)}^{2}R_{y}^{c}R_{y}^{c}{}^{H}$$

The above equation reveals that the estimation accuracy increases with the number of data blocks used *G* and the number of samples per data block *K*.

Compared with the matrix ${\hat{R}}_{y}^{c}$, greater concern is warranted for its EVD result. In subsequent processes, the eigenvalues were used to determine the signal subspace dimension *d* and detect a spoofing attack, and the eigenvectors were for the DOA estimation. Herein, the simulation results are provided to illustrate the influence of the value of *G* and *K* on the estimation accuracy of the signal number and DOA.

**Figure 6:**
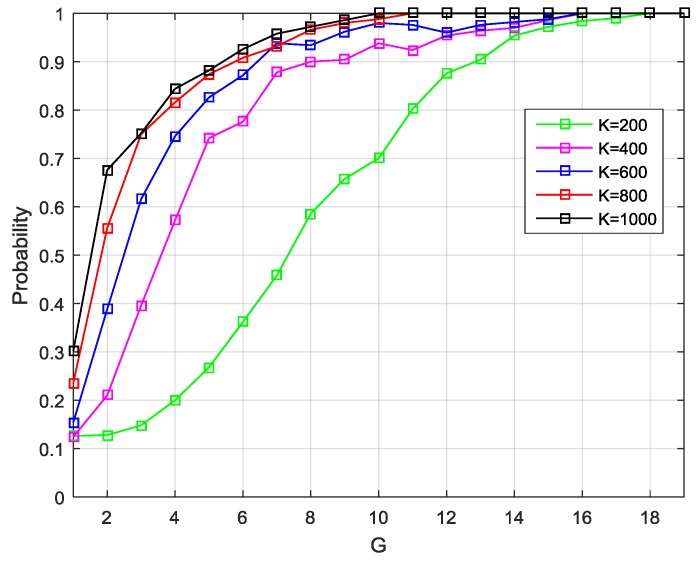
Probability of correct signal subspace dimension estimation versus G for different *K*.

**Figure 7:**
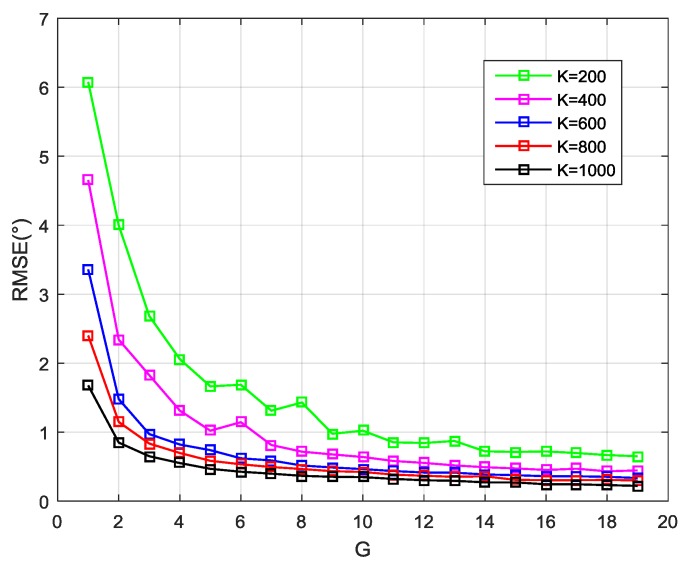
The root-mean-square-error (RMSE) of the directions-of-arrival (DOA) estimation versus G for different *K*.

For simplicity, the case is considered when there is no interference. Assume that four satellite signals are considered with the same power –157 dBW. The Monte Carlo simulations have been performed 1000 times, in which the DOAs of signals were changed randomly from 0° to 180°, while the initial phases of signals were selected randomly. In each trial, the cyclic correlation matrixes were estimated under different values of G and K to determine the signal subspace dimension and DOAs. Figure 6 shows the probability of correct signal subspace dimension estimation versus the data block number for the different sample number per data block. Figure 7 presents the root-mean-square-error (RMSE) of the DOA estimation results under the different values of G and K. The estimation accuracy of the subspace dimension can be seen, but also, the DOA estimation performance is shown to improve as G and K increase. When *G* is large enough, the performance gain of a larger *K* is not so obvious. However, the increase of G also means that more sample buffering is needed. Therefore, when this technique applies in the real system, the proper values of *G* and *K* should be selected according to the actual situation to achieve a compromise between algorithm performance and computational complexity.

### 4.2. Spoofing Detection Performance

In Section 3.2.3, the CCET algorithm was proposed to detect the presence of the spoofing attack. Herein, the performance of the proposed method is evaluated through simulations. These include: (1) The determination of the detection threshold with a given false alarm probability. (2) The influence of the number and power of spoofing signals on the detection probability.

#### 4.2.1. Determination of the Detection Threshold

Considering the situation of no spoofing attack, three groups of Monte Carlo simulations were performed to predict the PDF of the proposed test statistic under different signal numbers. The number of satellite signals was assumed to be $M^{A}=4,5,6$, respectively. In each group of simulations, two cases were considered. In one case, it was assumed that the power of each authentic signal was equal and set to be –157 dBW, which indicated the non-central parameter $\sigma_{0}=0$ in Equation (36). In the other case, the power of each signal was randomly chosen between –158 dBW to –156 dBW, which is more coincident with the real situations.

**Figure 8:**
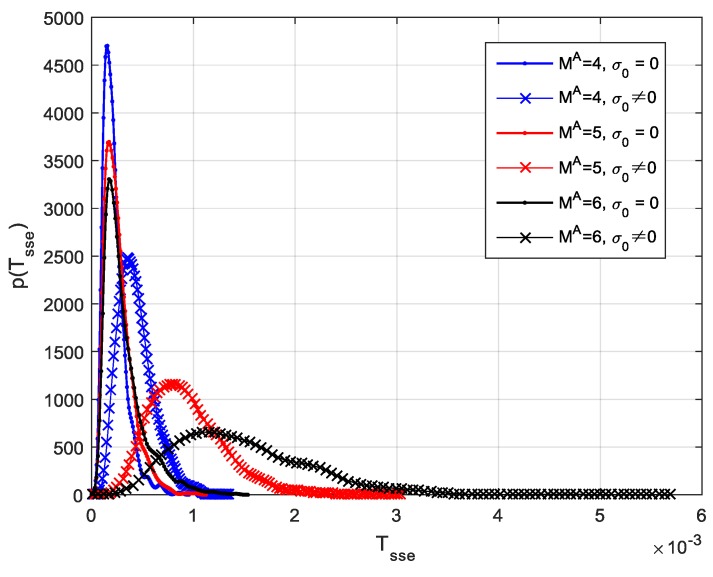
Empirical probability distribution functions (PDFs) of the *SSE* metrics for different signal numbers.

The empirical PDFs of the obtained SSE metrics in Equation (35) for different signal number are shown in Figure 8. Then, the detection threshold for a given false alarm probability can be calculated by Equation (39). Table 1 shows the threshold values corresponding to different $P_{fa}$ at different values of $M^{A},\;\sigma_{0}$.

#### 4.2.2. Probability of Spoofing Detection

Once the detection threshold values were determined, the next simulations were conducted to evaluate the probability of spoofing detection. It was assumed that the number of spoofing signals was equal to that of the authentic signals and each spoofing signal had the same PRN code as the corresponding authentic signal. The power of each authentic satellite was –157 dBW and the spoofing power varied from –163 dBW to –154 dBW. The code phase difference between each spoofing signal and their authentic counterpart was randomly chosen from 150 m to 600 m, and the Doppler frequency differences were all set as 10 Hz.

**Figure 9:**
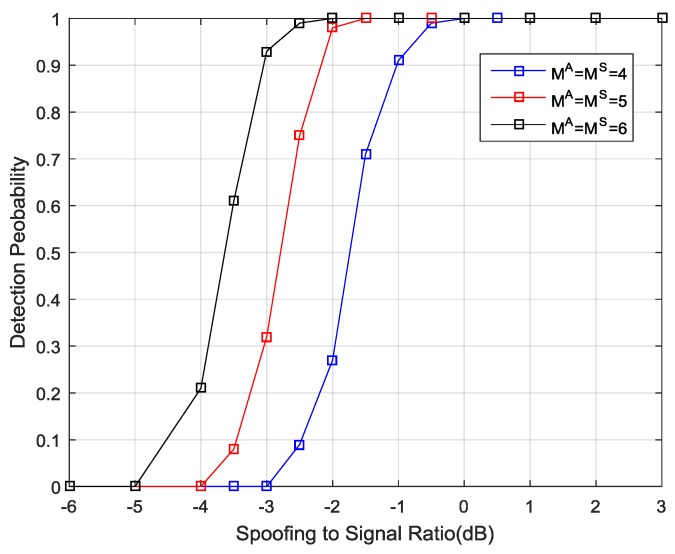
Spoofing detection probability with the power ratio of spoofing to the authentic signal.

In each trial of Monte Carlo simulations, the test statistic was calculated and compared to the predefined threshold value, which satisfies $P_{fa}<10^{-5}$. Figure 9 shows the probability of the spoofing detection as a function of the power ratio of spoofing to the authentic signal. It is observed that when the signal number is 4, 5, 6, the presence of spoofing signals starts to be detected as soon as the power ratio of spoofing to the authentic signal exceeds −3 dB, −4 dB, −5 dB, respectively. This is because the spoofing signals come from the same direction and the total power is higher than the power of each authentic signal. As the spoofing power increases, the detection performance of the proposed method increases as well. Notably, once the power of the spoofing signals exceeds the power of the authentic ones, the probability of spoofing detection in all scenarios exceeds 99%.

## 5. Simulation Results

In this section, more simulation results have been provided to demonstrate the effectiveness of the proposed interference suppression scheme in different application scenarios.

• Scenario 1:

In the first experiment, a uniform linear array was used, in which ten omnidirectional antennas were arranged in a straight line and the spacing between adjacent elements was half of a GPS L1 wavelength. Five authentic satellite signals PRN2, PRN5, PRN8, PRN19 and PRN26 were transmitted from the direction at the azimuth of −50°, −30°, 0°, 20° and 70° with the power assumed to be −157 dBW. There were two interference sources. One source transmitted five spurious signals PRN2, PRN5, PRN8, PRN19 and PRN26 from the direction at azimuth of 50°. The power of each spoofing signal was 3 dB higher than the authentic signal. The code phase differences between the spoofing signals and their authentic counterparts were all set as 150 m (about 0.5 chips) and the Doppler frequency differences were set as 10 Hz. The other source emitted the jamming signal from the direction at the azimuth of −5°. The jamming-to-signal power ratio (J/S) was assumed to be 60 dB. The additive Gaussian noise on each antenna was assumed to be white with spectral density −204 dBW/Hz. The bandwidth of the receiver, as well as the I/Q sampling frequency, was set to be 5 MHz. The recorded data length was 120 s and the proposed interference suppression scheme was executed every 1 second. The relevant results are as follows.

**Figure 10:**
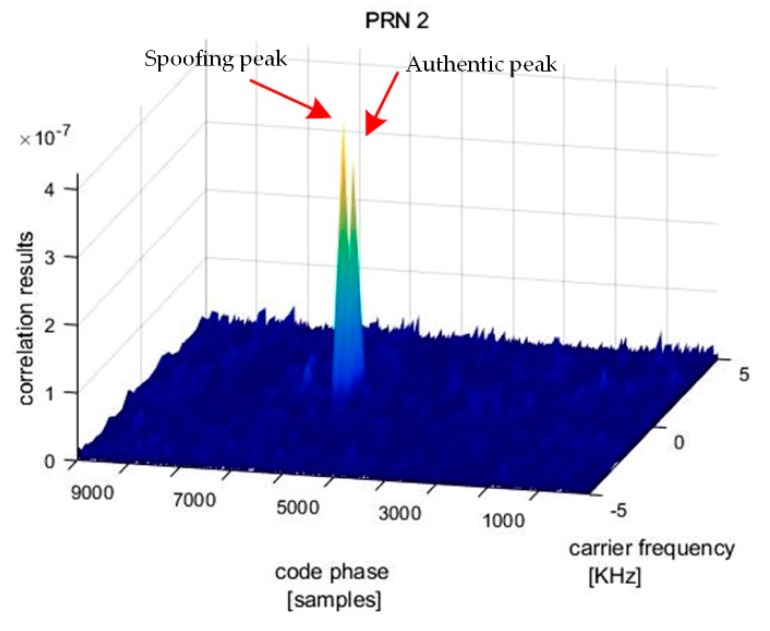
Correlation result of the receiver for PRN2 after jamming suppression.

After the subspace projection in the first stage, the output signal passed to the acquisition process of a GPS receiver to verify the jamming suppression effect. The acquisition result shows that five PRN signals are captured. For example, the correlation result for PRN2 is presented in Figure 10. It can be seen that there are two distinct correlation peaks, one for the authentic satellite signal and the other for the spoofing signal. It means that jamming has been removed from the received signal. As the spoofing signal has a higher power, a normal GNSS receiver can track it instead of the right signal.

In the spoofing detection and mitigation module, *G =* 9 data blocks were first selected, each of which contains *K =* 1000 samples, to estimate the cyclic correlation matrix. Then, the eigenvalues of this matrix are used to determine the number of signal sources based on the MDL criterion. For d∈{0,1,⋯,9}, the resulting values of the *MDL(d)* are shown in Table 2. The minimum of the MDL is obtained, as expected, at $\hat{d}=6$.

**Figure 11:**
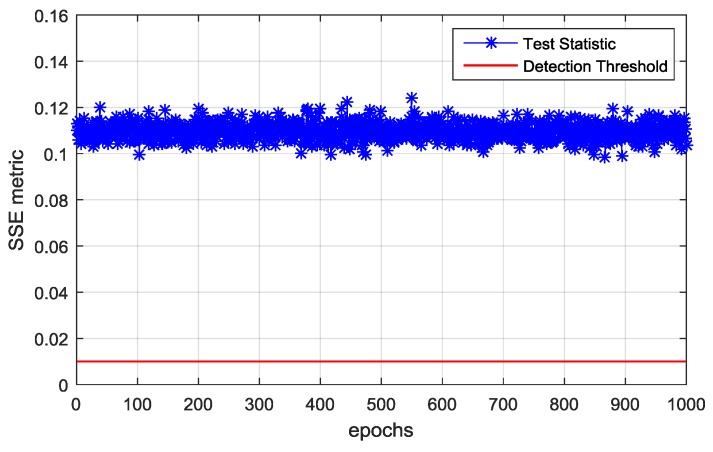
Comparison between the test statistic calculated by the cyclic correlation eigenvalue test (CCET) algorithm and the spoofing detection threshold.

**Figure 12:**
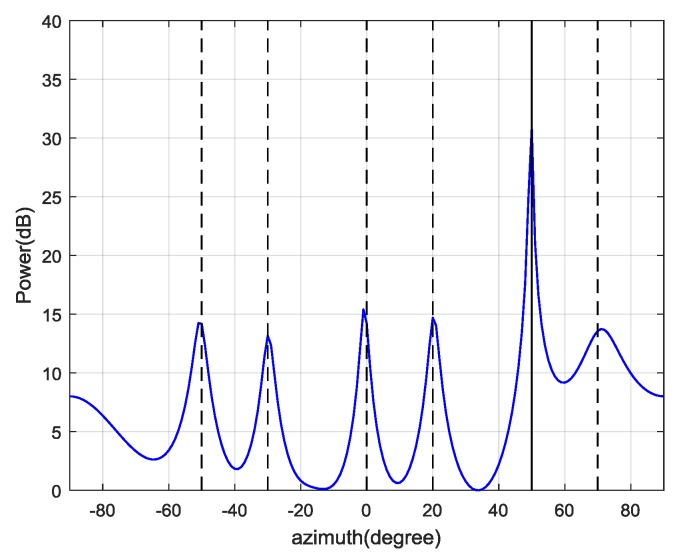
Estimated spatial spectrum of the jamming-free signal by Cyclic MUSIC algorithm.

Therefore, the first $\hat{d}=6$ eigenvalues are used for spoofing detection. Then, the CCET algorithm is used to calculate the test statistic, which is shown in Figure 11. It illustrates that the spoofing attack is successfully detected every epoch. Then, the first $\hat{d}=6$ eigenvectors construct the signal subspace to estimate the spatial power spectrum and the result is shown in Figure 12. The dashed lines represent the real DOAs of the authentic satellite signals, and the solid line represents the spoofing DOA. It shows that the Cyclic MUSIC algorithm can estimate the directions of all the signal sources effectively and the location of the maximum peak aligns with the spoofing DOA.

**Figure 13:**
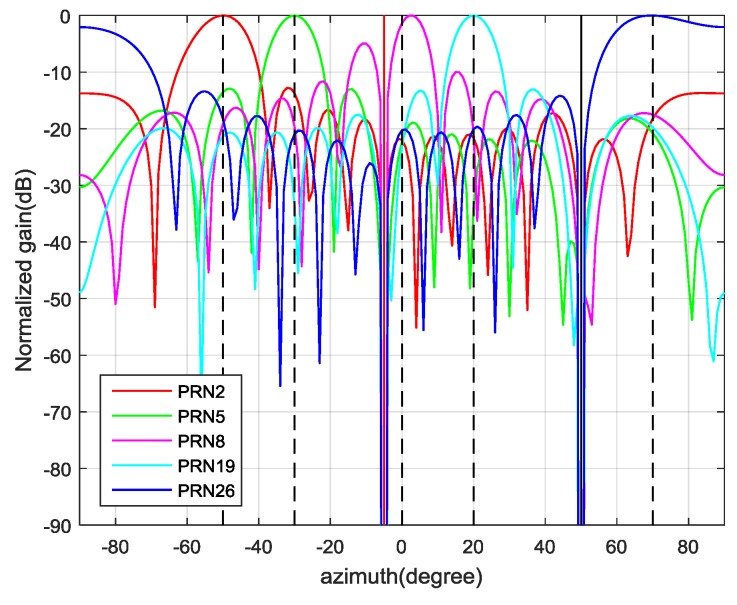
Beam pattern for each authentic satellite using obtained weight vectors.

**Figure 14:**
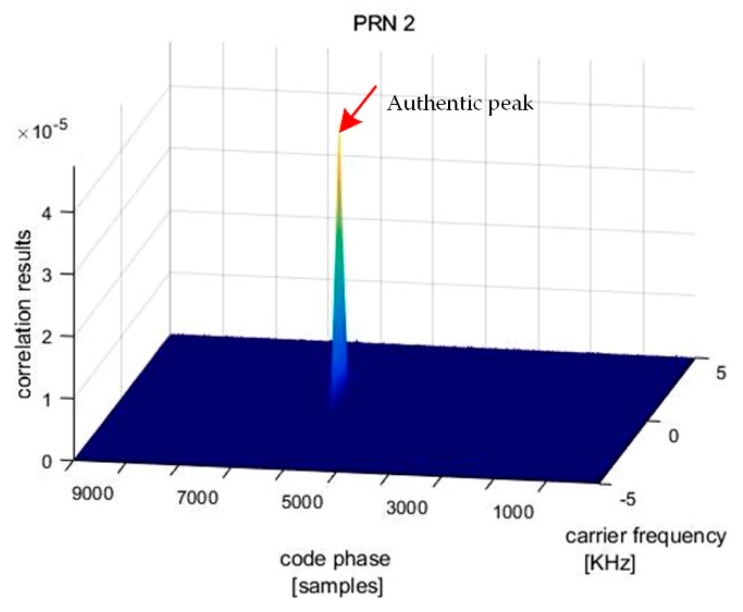
Correlation result for PRN2 after spoofing detection and mitigation.

Based on the above results, the final weight vector for each authentic satellite signal can be calculated by Equation (37) and the antenna beam patterns are shown in Figure 13. It demonstrates that the proposed interference suppression scheme can form nulls in the directions of spoofing and jamming, while the authentic satellite signal gets the maximum gain. Figure 14 shows the correlation result for PRN2 of the output signal, in which only the authentic peak is present.

• Scenario 2:

In Section 3, an alternative spoofing suppression scheme was provided when the number of array elements is less than the number of all the incoming signals (include jamming, spoofing and satellite signals). Herein, the feasibility of this method was verified by simulation. In this experiment, the ten-element ULA was still employed and the number of satellite signals was set to seven. Two jamming sources transmitted high power interferences from different directions. One spoofing source emitted seven spurious signals with the same PRNs as the authentic satellite. The PRN and DOA information of these signals are given in Table 3. The other parameters are the same as the values in Scenario 1.

**Figure 15:**
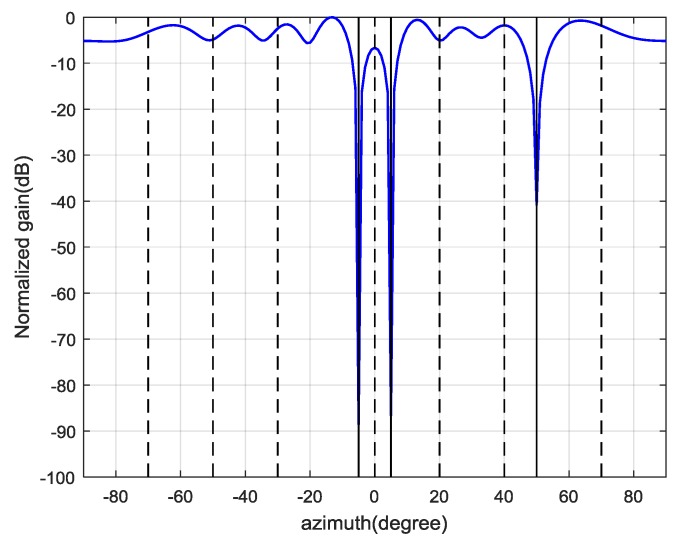
Quiescent beam pattern after jamming and spoofing mitigation.

The simulation results show that, in this scenario, the jamming signals can be detected and eliminated successfully in the first stage. In the spoofing detection module, the values of the *MDL(d)* are shown in Table 4. It can be seen that the *MDL(d)* is a monotonically decreasing function so that the number of signal sources cannot be determined. In this case, the eigenvector corresponding to the largest eigenvalue is regarded as the spoofing subspace and projects the array signal onto its null space. Figure 15 shows the final beam pattern after two projections. It turned out that the proposed method can eliminate jamming and spoofing signals in the case of a small array. Since the beamforming for each satellite cannot be performed, the authentic signals may be attenuated more or less.

• Scenario 3:

In the above two scenarios, a one-dimension line array was used to display the simulation results, such as the estimated spatial spectrum and the beam patterns, more intuitively. In order to verify that the proposed scheme is suitable for any arbitrary antenna array, in the next experiment, a 3 × 4 rectangular array was used, which consists of twelve omnidirectional antennas arranged as shown in Figure 2. Four authentic satellite signals were incident on the array from different directions. One jamming source transmitted the jamming signal and one spoofing source emitted four spurious signals from the same direction. Table 5 presents the DOAs of these signals in the form of the elevation and azimuth angles. The other parameters are set as the values in Scenario 1 and Scenario 2.

**Figure 16:**
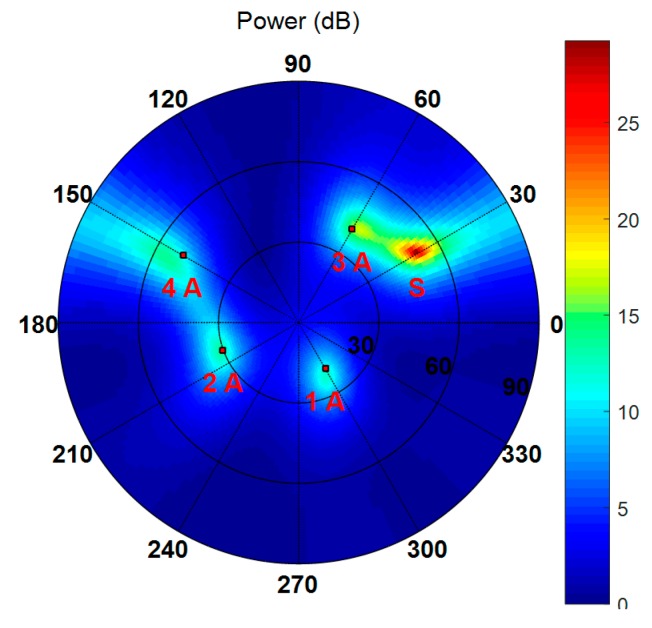
Spatial spectrum estimated by Cyclic MUSIC algorithm.

**Figure 17:**
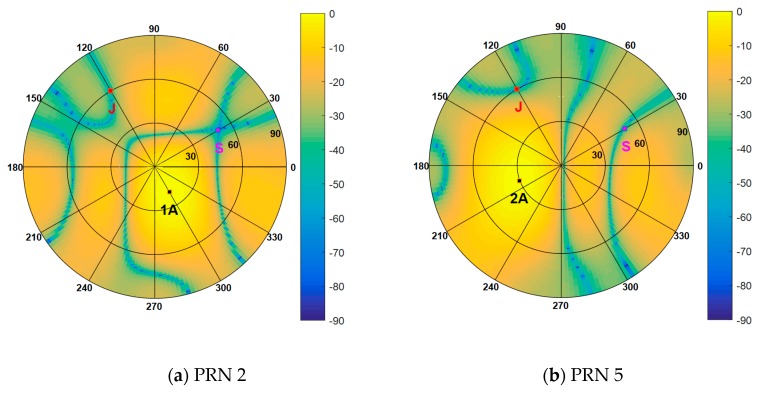

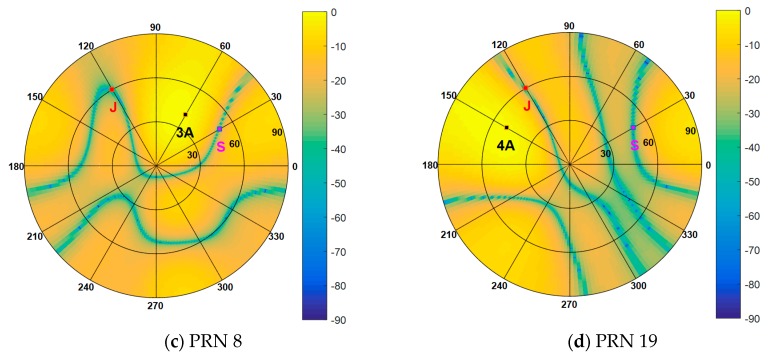
Beam patterns for each authentic satellite.

Figure 16 shows the spatial spectrum estimated by the Cyclic MUSIC algorithm in which the black dots denote the DOAs of all incident signals, S and A represent spoofing signal and authentic satellite signal, respectively. It indicates that the presence of spoofing interference can be detected and then mitigated through subspace projection and beamforming. Figure 17 shows the beam patterns for all the authentic satellites with respect to azimuth and elevation, in which J represents jamming. It can be seen that the weight vector calculated by the proposed method in this paper can suppress spoofing and jamming simultaneously and guarantee the gain of the authentic satellite signals. It can be concluded that the proposed interference suppression scheme is still valid when planar arrays are employed.

## 6. Conclusions

As the use of GNSS is pervasive in military and civil fields, interference like jamming and spoofing has shown its potential threats to modern GNSS applications. This paper introduces a two-stage GNSS interference suppression scheme based on antenna arrays. In the first stage, the subspace projection was adopted to remove the strong jamming signals. The second stage dealt with low power spoofing signals, in which the cyclostationarity of navigation signals was fully excavated to detect spoofing signals and estimated the spatial power spectrum before the despreading process. Then, the subspace projection mitigated the spoofing signals and beamforming for each satellite which ensured that the power of the authentic signals was not attenuated.

The simulation results show that the proposed scheme can detect jamming signals and form deep nulls (more than −90 dB) in beam patterns to eliminate them. When the code phase differences between the authentic and spoofing signals are more than 0.5 code chips, the scheme can detect the spoofing attack successfully and estimate the DOAs of all the signals accurately. The spoofing signals can be attenuated by more than 50 dB while the main-beam points to the desired satellite. It should be noted that our method is to distinguish between interference and satellite signals based on their differences in the spatial-domain. When the DOA of a satellite signal is close to the jamming’s or spoofing’s DOA, this signal can be eliminated in interference nulls. Fortunately, according to the geometry distribution of the GPS satellites, there are not many authentic signals from the direction close to the interference DOA.

However, in the spoofing scenario of a small time-offset, the correlation between the authentic and spoofing signals may cause poor DOA estimation performance. Given this problem, the forward-backward spatial smoothing techniques for de-correlation can be used to improve the DOA estimation performance, but it may also result in the loss of array freedom. When both the satellites and receiver are moving, the calculation of the cyclic correlation matrix over a long data set may provide the necessary smoothing needed. The authors intend to make further investigations in future work.

## Figures and Tables

**Table 1:** Detection thresholds for given $P_{fa}$ with different $M^{A},\;\sigma_{0}$.

		$P_{fa}=10^{-1}$	$P_{fa}=10^{-2}$	$P_{fa}=10^{-3}$	$P_{fa}=10^{-4}$	$P_{fa}=10^{-5}$
$M^{A}=4$	$\sigma_{0}=0$	$0.38\times 10^{-3}$	$0.66\times 10^{-3}$	$0.87\times 10^{-3}$	$0.91\times 10^{-3}$	$0.94\times 10^{-3}$
	$\sigma_{0}\neq 0$	$0.68\times 10^{-3}$	$0.96\times 10^{-3}$	$1.15\times 10^{-3}$	$1.29\times 10^{-3}$	$1.32\times 10^{-3}$
$M^{A}=5$	$\sigma_{0}=0$	$0.45\times 10^{-3}$	$0.73\times 10^{-3}$	$1.01\times 10^{-3}$	$1.11\times 10^{-3}$	$1.11\times 10^{-3}$
	$\sigma_{0}\neq 0$	$1.44\times 10^{-3}$	$2.12\times 10^{-3}$	$2.64\times 10^{-3}$	$2.89\times 10^{-3}$	$2.98\times 10^{-3}$
$M^{A}=6$	$\sigma_{0}=0$	$0.61\times 10^{-3}$	$1.04\times 10^{-3}$	$1.38\times 10^{-3}$	$1.48\times 10^{-3}$	$1.52\times 10^{-3}$
	$\sigma_{0}\neq 0$	$2.41\times 10^{-3}$	$3.45\times 10^{-3}$	$4.99\times 10^{-3}$	$5.46\times 10^{-3}$	$5.64\times 10^{-3}$

**Table 2:** The corresponding values of the *MDL* function for different *d*.

*d*	0	1	2	3	4	5	6	7	8	9
*MDL(d)*	801.3	673.7	553.7	424.1	356.8	314.1	298.7	320.8	335.5	345.3

**Table 3:** Simulation parameters of the signal sources.

	Sat1	Sat2	Sat3	Sat4	Sat5	Sat6	Sat7	Spoofing	Jam1	Jam2
PRN	2	5	8	19	21	26	29	[2,5,8,19,21,26,29]		
DOA	−50°	−30°	0°	20°	40°	70°	−70°	50°	−5°	5°

**Table 4:** The values of the *MDL(d)* in the case of a small array.

*d*	0	1	2	3	4	5	6	7	8
*MDL(d)*	1033.5	633.1	625.7	615.1	603.8	584.7	566.8	557.5	475.9

**Table 5:** Simulation parameters of the signal sources.

	Sat1	Sat2	Sat3	Sat4	Spoofing	Jamming
PRN	2	5	8	19	[2,5,8,19]	
DOA	(20°,300°)	(30°,200°)	(40°,60°)	(50°,150°)	(50°,30°)	(60°,120°)
